# The Plasma Kallikrein–Kininogen Pathway Is Critical in the Pathogenesis of Colitis in Mice

**DOI:** 10.3389/fimmu.2018.00021

**Published:** 2018-02-06

**Authors:** Bo Wang, Aizhen Yang, Zhenzhen Zhao, Chao He, Yuanyuan Liu, Robert W. Colman, Jihong Dai, Yi Wu

**Affiliations:** ^1^Cyrus Tang Hematology Center, Collaborative Innovation Center of Hematology, Soochow University, Suzhou, China; ^2^The Sol Sherry Thrombosis Research Center, Temple University School of Medicine, Philadelphia, PA, United States; ^3^Department of Pathology and Laboratory Medicine, Rutgers New Jersey Medical School, Newark, NJ, United States

**Keywords:** kallikrein–kinin system, colitis, inflammation, neutrophil, cytokine

## Abstract

The kallikrein–kinin system (KKS) consists of two serine proteases, prekallikrein (pKal) and factor XII (FXII), and a cofactor, high-molecular-weight kininogen (HK). Upon activation of the KKS, HK is cleaved to release bradykinin. Although the KKS is activated in humans and animals with inflammatory bowel disease (IBD), its role in the pathogenesis of IBD has not been characterized. In the present study, we determined the role of the KKS in the pathogenesis of IBD using mice that lack proteins involved in the KKS. In two colitis models, induced by dextran sulfate sodium (DSS) or 2,4,6-trinitrobenzene sulfonic acid (TNBS), mice deficient in HK, pKal, or bradykinin receptors displayed attenuated phenotypes, including body weight loss, disease activity index, colon length shortening, histological scoring, and colonic production of cytokines. Infiltration of neutrophils and inflammatory monocytes in the colonic lamina propria was reduced in HK-deficient mice. Reconstitution of HK-deficient mice through intravenous injection of HK recovered their susceptibility to DSS-induced colitis, increased IL-1β levels in the colon tissue and bradykinin concentrations in plasma. In contrast to the phenotypes of other mice lacking other proteins involved in the KKS, mice lacking FXII had comparable colonic inflammation to that observed in wild-type mice. The concentration of bradykinin was significantly increased in the plasma of wild-type mice after DSS-induced colitis. *In vitro* analysis revealed that DSS-induced pKal activation, HK cleavage, and bradykinin plasma release were prevented by the absence of pKal or the inhibition of Kal. Unlike DSS, TNBS-induced colitis did not trigger HK cleavage. Collectively, our data strongly suggest that Kal, acting independently of FXII, contributes to experimental colitis by promoting bradykinin release from HK.

## Introduction

Inflammatory bowel diseases (IBDs), such as ulcerative colitis (UC) and Crohn’s disease (CD), are debilitating disorders caused by gastrointestinal mucosal damage and inflammation. The etiology and pathogenesis of IBDs remain unknown; however, they are likely triggered through a complex interplay between genetic susceptibility, host immune system dysregulation, and environmental factors (1). In general, IBDs are associated with a compromised gastrointestinal barrier, leading to inflammation directly through tissue injury and indirectly *via* the production of various pro-inflammatory mediators that recruit immune cells. Plasma proteolytic cascades have been also reported to be important in IBD development (2).

The plasma kallikrein–kinin system (KKS) consists of a group of plasma proteins that responds to pathophysiological stimuli and tissue injury (3), specifically two serine proteinases [coagulation factor XII (FXII) and prekallikrein (pKal)], and a non-enzymatic cofactor [high-molecular-weight kininogen (HK)] (4). KKS proteins interact with many physiologic and pathophysiologic systems, such as the immune and complement systems (3). The KKS cascade is activated when FXII and HK assembled on negatively charged surfaces. Following autocatalysis, FXIIa cleaves pKal, generating Kal, which in turn activates additional FXII zymogens. Activated Kal, which is the major bradykinin-releasing enzyme in blood, cleaves HK to HKa and bradykinin, a short-lived pro-inflammatory nanopeptide (5). pKal can also be activated by the lysosomal enzyme prolylcarboxypeptidase and heat-shock protein 90 independently of FXII (6–9). Bradykinin can be hydrolyzed by carboxypeptidase N (in plasma) or carboxypeptidase M (on endothelial cells) to produce des(Arg9)-bradykinin. Bradykinin and des(Arg9)-bradykinin are the agonists of two G protein-coupled receptors (designated as B2R and B1R, respectively) (10, 11).

Activation of the KKS has been documented in patients with active UC and CD (12, 13) and in rat models of acute colitis (14, 15). A significant decrease in pKal and HK levels was observed in plasma of patients with active UC (12, 13), presumably reflecting increased KKS activation/consumption. Kal immunoreactivity was stronger in the intestines of patients with UC and CD than in those of normal controls, suggesting that pKal might activate the KKS extravascularly following extravasation/diffusion of plasma through injured intestinal tissues (13). B1R and B2R are expressed in the intestines of healthy individuals and patients with UC and CD, but their expression levels are significantly increased in active UC and CD intestines (16). In a genetically susceptible strain of Lewis rats with granulomatous enterocolitis induced by peptidoglycan–polysaccharide polymers from group A streptococci, consumption of pKal and HK proteins was closely correlated with chronic intestinal inflammation (17, 18). In a distinct indomethacin-induced enterocolitis model, Lewis rats also displayed KKS activation (19), and in the human leukocyte antigen-B27 transgenic rat model of human IBD, a monoclonal antibody against HK decreased clinical scores and colonic lesions of pre-existing IBD (20). Another disease model, in which IBD was induced in Sprague-Dawley rats by dextran sulfate sodium (DSS), demonstrated that the B2R antagonist FR173657 inhibited shortening of the colon, suggesting that KKS activation worsens colitis due to bradykinin-induced B2R signaling (21).

Although these observations in humans and rats suggest that KKS activation can aggravate the pathology of IBD, the respective roles of pKal, FXII, HK, and kinin-signaling pathways have not been systematically characterized. In the present study, this issue was addressed using two models of experimental colitis, induced by DSS or 2,4,6-trinitrobenzene sulfonic acid (TNBS), in transgenic mouse strains lacking HK, pKal, FXII, or B2R/B1R. Our results link the pathological outcome of DSS or TNBS-induced colitis to kinin release *via* the pKal/HK pathway. Intriguingly, FXII-deficient mice remained susceptible to colitis, suggesting that activation of the KKS in the inflamed mucosa occurs through a pathway independent of this factor and contact system-induced coagulation.

## Materials and Methods

### Mice

*Klkb1*^−/−^ mice with a disrupted pKal gene were generated by transcription activator-like effector nucleases (TALEN)-mediated targeted gene disruption, with the assistance of Cyagen Bioscience, Inc. (Santa Clara, CA, USA). The constructed TALEN repeats were designed to bind to exon 4 of the *Klkb1* gene and were subcloned into a mammalian expression vector, pCMV-TALEN. Poly A-tailed mRNAs were produced using the Ambion mMessage mMachine kit for microinjection (Foster City, CA, USA). *In vitro*-transcribed mRNA encoding this TALEN pair was microinjected into fertilized eggs from C57BL/6 mice. Approximately 30 injected zygotes were transferred into the fallopian tubes of each recipient mouse. A 25-base deletion (GAGCATTACAGGGACTTTGCCAAGA; 243–267 of the open reading frame) at exon 4 was detected in the C57BL/6 background of the resulting offspring, which were selected for further breeding and characterization. This 25-base deletion caused a frameshift and created an early stop codon, resulting in truncated *Klkb1* (pKal) after Leu92. Offspring were identified by genotyping *via* tail-biopsied DNA using the following primers in a 35-cycle PCR reaction: P1: 5′-GCATTACAGGGACTTTGCC-3′, P2: 5′-TCTTTCTTGGTGGTCTCGTC-3′; P3:5′-GGTTGCTTCATGAAAGAAT-3′, P4: 5′-GGCTGAGCACTATAACTG-3′. These primer pairs were designed to yield PCR products derived from the wild-type and 25 bp-deleted alleles. RNA was extracted from the mouse liver using TRIzol (Invitrogen, Carlsbad, CA, USA). Total RNA (2 µg) was used for first-strand cDNA synthesis using oligo(dT)_15_ and Superscript II RNase H^−^ Transcriptase (Invitrogen). The product of the first-strand reaction was amplified by PCR using primers P1 (indicated above); P5: 5′-GGTGTTCCGCTTGCACTG-3′; and β-actin (F: 5′-GTCCCTCACCCTCCCAAAAG-3′, R: 5′-GCTGCCTCAACACCTCAACCC-3′), to ensure that equal amounts of cDNA were added to each reaction. Products were analyzed on 2% agarose gels. To verify the absence of the pKal protein, plasma from wild-type and *Klkb1*^−/−^ mice was analyzed by immunoblotting with a goat polyclonal anti-pKal antibody (R&D Systems, Minneapolis, MN, USA) and a secondary donkey anti-goat antibody conjugated with DyLight 800 (Li-Cor, Lincoln, Nebraska, USA). Homozygous *Klkb1*-deficient (*Klkb1*^−/−^) mice were viable, healthy, fertile, born with normal Mendelian frequency and did not display any gross physical or behavioral abnormalities.

Mice lacking HK (*Kng1*^−/−^) were generated in our previous studies (22). Double knockout mice lacking both bradykinin receptors (*B1RB2R*^−/−^) were purchased from the Jackson Laboratory (Bar Harbor, ME, USA). Mice lacking Factor XII (*FXII*^−/−^) were kindly provided by Dr. F. Castellino from the University of Notre Dame. All knockout mice, including *Kng1*^−/−^ and *Klkb1*^−/−^ mice, were backcrossed on a C57BL/6 background for more than 10 generations. Mice were maintained in a pathogen-free facility and monitored in accordance with the guidelines of the Institutional Animal Care and Use Committee.

### Induction and Assessment of DSS-Induced Colitis

Mice used for the induction of colitis with DSS and TNBS were gender- and age-matched, with 8-week-old littermates of both genders in a ratio of 1:1. Treatment and data acquisition, including scoring, were performed in a blinded manner. For DSS-induced colitis, mice were treated with 2.5% (wt/vol) DSS (molecular mass = 36–50 kDa; MP Biomedicals, Santa Ana, CA, USA) in drinking distilled water (DW) *ad libitum* for 8 days with DW alone for the control group (23). The amount of DSS consumed by each mouse was recorded. There were no differences in intake between experimental groups. Mice were weighed every day to determine percentage weight changes, which was calculated as percentage body weight change (weight at day *x*/weight at day 0 × 100). The animals were monitored clinically for rectal bleeding, diarrhea, and other general health signs, including hunched posture and failure to groom. The disease activity index (DAI) was assessed by the combined score of weight loss, stool consistency, and bleeding. Scores were defined as follows: stool consistency was graded 0 for no diarrhea, 2 for loose stool that did not stick to the anus, and 4 for liquid stool that stuck to the anus. The presence of fecal blood was graded 0 for none, 2 for moderate, and 4 for gross bleeding. For weight loss evaluation, a value of 0 was assigned if the body weight remained within 1% or higher of baseline, 1 for a 1–5% loss, 2 for a 5–10% loss, 3 for a 10–15% loss, and 4 for greater than 15% loss. For histological observations, the colons were isolated, flushed with PBS, and fixed in 4% formaldehyde solution. Tissues were embedded in paraffin, sectioned, mounted on glass slides, and deparaffinized. H&E-stained paraffin section images were acquired, followed by pathological evaluation in a blinded manner and scored as described previously (24). Briefly, the severity of inflammation (0–3: none, slight, moderate, severe); depth of injury (0–3: none, mucosal, mucosal and submucosal, transmural); and crypt damage (0–4: none, basal 1/3 damaged, basal 2/3 damaged, only surface epithelium intact, entire crypt, and epithelium loss) were monitored. The score of each parameter was multiplied by a factor reflecting the percentage of tissue involvement (1–4: 0–25, 26–50, 51–75, and 76–100%, respectively) and summed. White blood cell count and partial thromboplastin time (APTT) were measured as previously described (25).

### Induction and Assessment of TNBS-Induced Colitis

According to a previously described methodology (23), 5% TNBS (Sigma-Aldrich, St. Louis, MO, USA) solution mixed 1:1 (v/v) with absolute ethanol (EtOH) was applied intrarectally to mice. Control mice received 50% EtOH. Histological scoring was based on a semiquantitative scoring system where features were graded as follows: extent of destruction of normal mucosal architecture (0: normal; 1, 2, and 3: mild, moderate, and extensive damage, respectively); presence and degree of cellular infiltration (0: normal; 1, 2, and 3: mild, moderate, and transmural infiltration, respectively); extent of muscle thickening (0: normal; 1, 2, and 3: mild, moderate, and extensive thickening, respectively); presence or absence of crypt abscesses (0: absent; 1: present); and presence or absence of goblet cell depletion (0: absent; 1: present). The scores for each feature were summed, with a maximum possible score of 11.

### Isolation of Colon Lamina Propria (LP) Cells and Flow Cytometric Analysis

Intestinal LP cells were obtained following digestion of the colon in RPMI containing 5% fetal calf serum, 5 mM EDTA, and 2 mM dithiothreitol (26). Briefly, the colons were minced, the pieces were incubated with agitation in digestion buffer for 30 min and filtered. The retained fraction was digested in RPMI containing 5% fetal calf serum, 400 U/mL of type-I collagenase (Thermo Scientific, Waltham, MA, USA), and 0.1 mg/mL of DNase I (Takara Bio, Inc., Shiga, Japan) and filtered. The flow-through was centrifuged, and the cells were stained using FITC-conjugated anti-CD45, PE-conjugated anti-CD11b, and PerCP-conjugated Gr-1 (eBioscience, San Diego, CA, USA) (27), for flow cytometric analysis.

### Enzyme-Linked Immunosorbent Assay (ELISA)

Part of each colon was homogenized mechanically in PBS containing 1% NP-40 and complete protease inhibitor cocktail containing AEBSF, Aprotinin, Bestatin, E-64, Leupeptin, and Pepstatin A (I3786, Sigma-Aldrich). Mouse cytokines (R&D Systems) and myeloperoxidase (MPO) (USCN Life Science, Wuhan, China) were measured by ELISA. Plasma bradykinin concentrations were determined using an ELISA kit (Enzo Life Science, Farmingdale, NY, USA).

### Chromogenic Assay of Kal and FXIIa Activity

Kal activity was spectrophotometrically monitored *via* conversion of the chromogenic substrate H-D-Pro–Phe–Arg-pNA·2HCl, S-2302 (0.5 mM; HYPHEN BioMed, Neuville-sur-Oise, France) using a Bio-Kinetics Reader (BioTek Instruments, Inc., Winooski, VT, USA), as described previously (28, 29). To determine the specific effect of DSS on the activation of Kal, a monoclonal antibody (IgG1) against Kal M0202-H03 (Dyax, now a part of Shire, Burlington, MA, USA) was used, and an isotype-matched IgG1 (Sigma) was used as a control. Human and mouse plasma was prepared using sodium citrate as coagulant. In brief, 90 µL of diluted plasma (1:1) was preincubated with 10 µL of antibody or control buffer for 30 min. Ninety microliters of pretreated plasma were incubated with 10 µL of DSS for 10 min. Ten microliters of DSS-treated plasma were added to 90 µL of substrate in 96-well plate. A chromogenic substrate of FXIIa (SPECTROZYME^®^, Sekisui Diagnostics, Lexington, MA, USA) was used to measure FXIIa generation. The high sensitivity and specificity for FXIIa was demonstrated in a previous study (30). The positive control for FXII activation was 100 µg/mL of kaolin. The optical density of 0.5 mM substrate hydrolysis was measured at 405 nm using a spectrophotometer (SpectraMax M5, Molecular Devices, Sunnyvale, CA, USA) (31).

### Western Blotting

To identify activated/cleaved components of the KKS, platelet-free human and mouse plasma were prepared using EDTA as an anticoagulant. After centrifugation at 1,000 × *g* for 10 min, plasma was collected and incubated with DSS for 30 min, before the addition of Laemmli loading buffer containing 2-mercaptoethanol to stop the reactions. Cleavage of the KKS proteins was analyzed by immunoblotting using a rabbit polyclonal anti-HK antibody (Abgent, Inc., San Diego, CA, USA) which can detect the heavy chain of both human and mouse HK, a rabbit monoclonal anti-pKal antibody for recognizing human pKal (Abcam, Cambridge, MA, USA), a goat polyclonal anti-pKal antibody for recognizing mouse pKal (R&D Systems, Minneapolis, MN, USA) and a monoclonal anti-FXII antibody for recognizing human FXII (B7C9, ThermoFisher Scientific, Waltham, MA, USA).

### Real-time RT-PCR and PCR

Total RNA was extracted using TRIzol (Invitrogen). cDNA was synthesized with the RevertAid First-Strand cDNA Synthesis Kit (Thermo Scientific). Quantitative real-time PCR was performed using the Maxima SYBR Green/ROX qPCR Master Mix kit (Fermentas, Vilnius, LT, USA) according to the manufacturer’s instructions. mRNA levels were normalized to the levels of β-actin. Primer pairs used are as follows: interleukin-1β (IL-1β) F: 5′-TGTGTCTTTCCCGTGGACCT-3′, R: 5′-CAGCTCATATGGGTCCGACA-3′; IL-6 F: 5′-GGTGACAACCACGGCCTTCCC-3′, R: 5′-AAGCCTCCGACTTGTGAAGTGGT-3′; interferon-γ F: 5′-GCTCTGAGACAATGAACGCTAC-3′, R: 5′-TCTTCCACATCTATGCCACTTG-3′; tumor necrosis factor (TNF)-α F: 5′-GCCTCTTCTCATTCCTGCTTG-3′, R:5′-CTGATGAGAGGGAGGCCATT-3′; β-actin F: 5′-GTGCTATGTTGCTCTAGACTTCG-3′, R: 5′-ATGCCACAGGATTCCATACC-3′.

### Statistical Analysis

GraphPad Prism software was used for statistical evaluation (GraphPad, Inc., Chicago, IL, USA). The data were expressed as the mean ± SEM of at least three independent experiments, unless otherwise indicated. For parametric comparison, one-way analysis of variance (ANOVA) followed by Tukey’s test for multiple groups, or a two-tailed, unpaired Student’s *t*-test for two groups were used. For nonparametric comparison, a Mann–Whitney test was used. A *P* < 0.05 was considered statistically significant.

## Results

### *Kng1*^−/−^ Mice Are Protected against DSS-Induced Colitis

**Figure 1:**
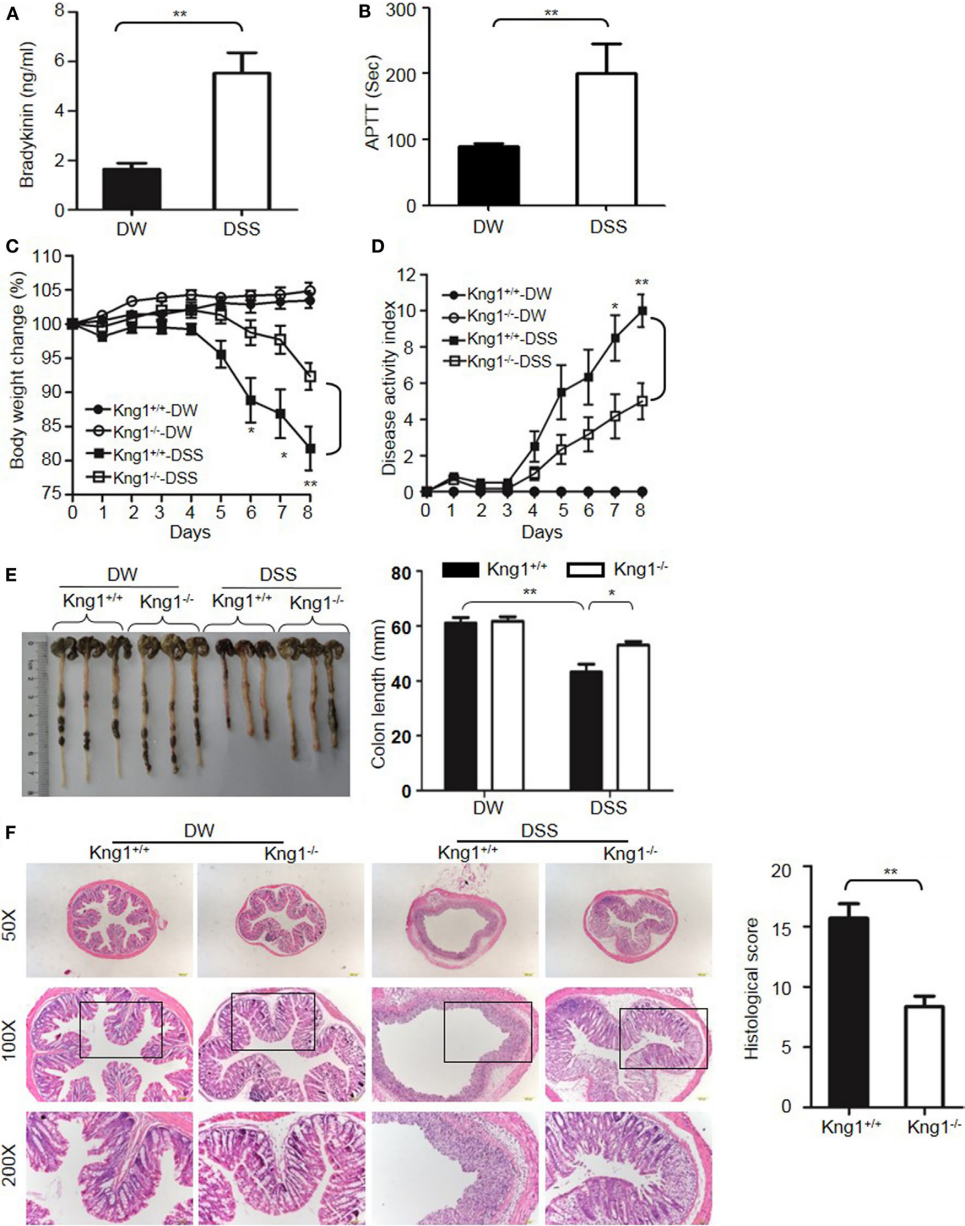
Kng1 deficiency ameliorates dextran sulfate sodium (DSS)-induced colitis. **(A,B)** WT C57BL/6 mice were treated with distilled water (DW) or 2.5% DSS for 8 days. Bradykinin levels **(A)** and APTT **(B)** were measured in the plasma. **(C–E)** Four groups of *Kng1*^+/+^ mice and *Kng1*^−/−^ mice (*n* = 12) were treated with DW or 2.5% DSS for 8 days. Body weight change **(C)** and disease activity index **(D)** were monitored daily. Body weight change was compared with body weight on day 0. On day 8, the cecum and colon were collected. The representative gross appearance of the colon is shown here [left panel, **(E)**]. Colon length was measured [right panel, **(E)**]. **(F)** The distal colon was processed for histological H&E staining. Representative images (left panel) and histological scoring (right panel) are shown. Statistical significance was determined using Student’s *t*-test **(A,F)**, one-way analysis of variance **(C,E)** or Mann–Whitney test **(B,D)**: *P < 0.05; **P < 0.01; ***P < 0.001.

The DSS-induced colitis model is often used in IBD research because of its rapidity, simplicity, reproducibility, and controllability. In DSS-treated WT mice, bradykinin levels and APTT, an index of intrinsic coagulation activity, were significantly increased compared to control mice treated with DW (Figures 1A,B), suggesting activation of the KKS and release of bradykinin in this model. To determine the role of HK in DSS-induced colitis, we generated a mouse strain with a disrupted gene for *Kng1*, which encodes HK. On day 8 after DSS administration, control *Kng1*^+/+^ mice developed severe colitis, with symptoms including body weight loss, loose stool consistency, and gross bleeding, as demonstrated by the DAI compared to that of DW-treated mice (Figures 1C,D). DSS-treated *Kng1*^+/+^ mice also had a significantly shortened colons (Figure 1E). Histological analysis of the colons from *Kng1*^+/+^ mice revealed loss of epithelial integrity and crypts, submucosal edema, and intense infiltration of inflammatory cells, as well as a remarkable increase in the histological score (Figure 1F). In contrast, DSS-treated *Kng1*^−/−^ mice had significant amelioration of colitis development and severity, as indicated by body weight, DAI, colon length, and histological changes (Figures 1C–F), suggesting that HK is required for the pathogenesis of DSS-induced colitis.

**Figure 2:**
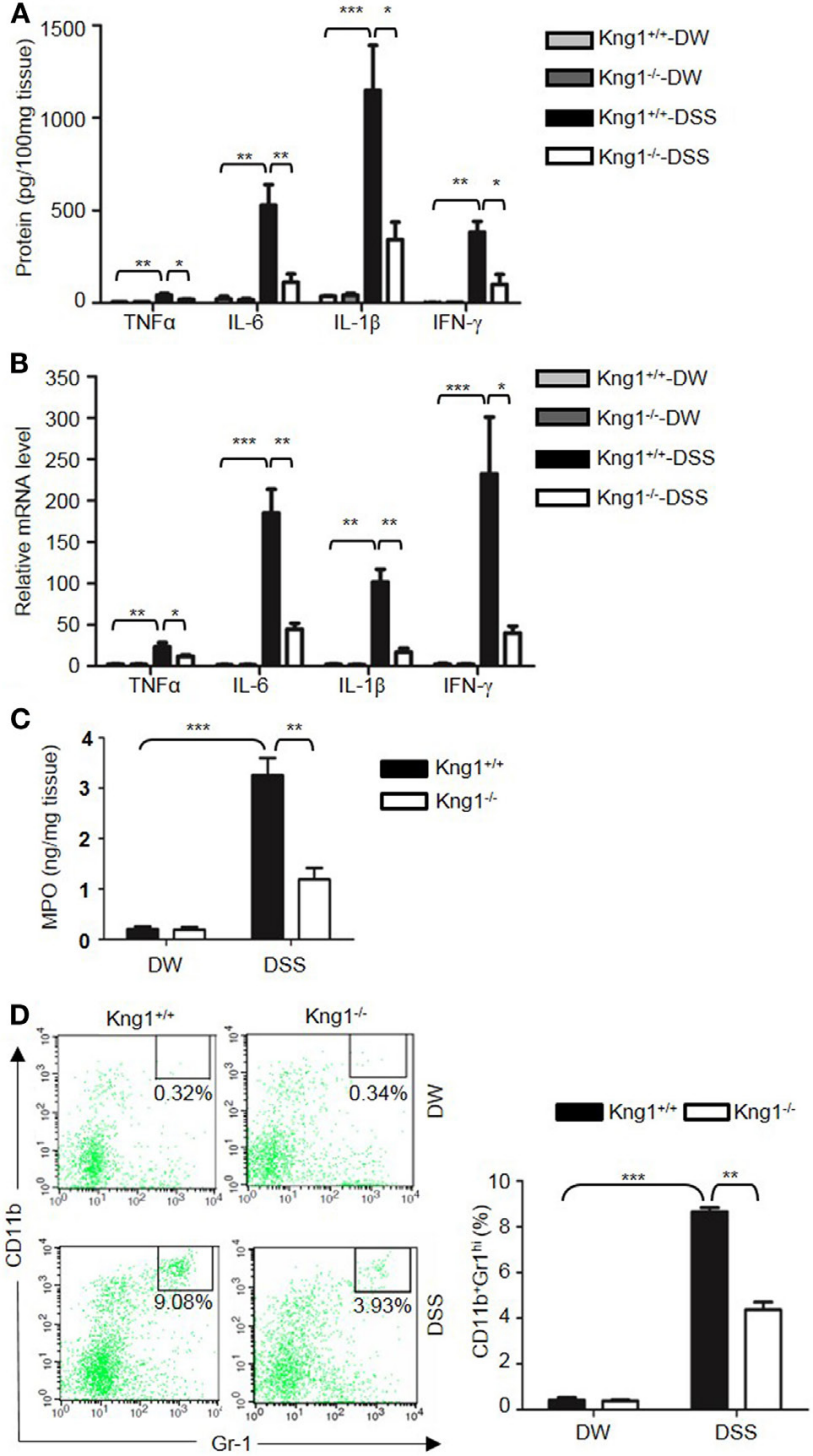
Kng1 deficiency reduces cytokine production and recruitment of neutrophil and inflammatory monocytes to inflamed colonic tissue. Four groups of *Kng1*^+/+^ mice and *Kng1*^−/−^ mice (*n* = 12) were treated with distilled water (DW) or 2.5% dextran sulfate sodium (DSS) for 8 days. **(A)** Cytokine levels in the colon homogenates were measured by enzyme-linked immunosorbent assay (ELISA). **(B)** RNA was isolated from the colon and cytokine expression levels were measured by real-time RT-PCR. **(C)** Myeloperoxidase (MPO) concentrations were measured in colon homogenates using ELISA. **(D)** Lamina propria cells were isolated from the colon, and the percentage of CD45^+^CD11b^+^Gr-1^Hi^ cells was assessed by flow cytometry. Statistical significance was determined by one way analysis of variance. *P < 0.05; **P < 0.01; ***P < 0.001.

Genetic studies have shown that inflammatory cytokines, such as TNFα, IL-1β, IL-6, and IFN-γ, are elevated in colitis, and are important in the development of mucosal inflammation (32–35). We, therefore, determined if HK regulates the production of these cytokines. On day 8 after DSS treatment, the concentration of TNFα, IFN-γ, IL-1β, and IL-6 were significantly increased in the colon homogenate of DSS-treated WT mice compared to in DW-treated WT mice (Figure 2A). In contrast, these cytokine levels were significantly lower in DSS-treated *Kng1*^−/−^ mice than in WT mice (Figure 2A). Moreover, the mRNA levels of TNFα, IFN-γ, IL-1β, and IL-6 were upregulated in DSS-treated WT mice and significantly downregulated in DSS-treated *Kng1*^−/−^ mice (Figure 2B). These observations suggest that HK is involved in cytokine production in DSS-induced colitis.

Infiltration of neutrophils into the intestinal mucosa is a pathological hallmark of active IBD, which contributes to tissue damage. Abundant infiltrated neutrophils have been detected in the colonic LP and epithelial layer of human patients with IBD, and in experimental mouse colitis models (36, 37). Since MPO is abundantly expressed in neutrophils, we measured MPO concentration in the colon homogenate to represent neutrophil recruitment (38, 39). DSS treatment significantly increased MPO levels in WT mice (Figure 2C); however, the MPO level in DSS-treated *Kng1*^−/−^ mice was significantly lower than in WT mice (Figure 2C). To quantify the recruitment of neutrophils and inflammatory monocytes, LP cells were analyzed by flow cytometry. Neutrophils and inflammatory monocytes, defined as CD11b^+^Gr-1^hi^ cells (Figure 2D, left panel) were significantly increased in DSS-treated WT mice, while the number of these cells was reduced in DSS-treated *Kng1*^−/−^ mice (Figure 2D, right panel). These results indicate that in the absence of HK, the infiltration of inflammatory cells into the gut mucosa decreases.

**Figure 3:**
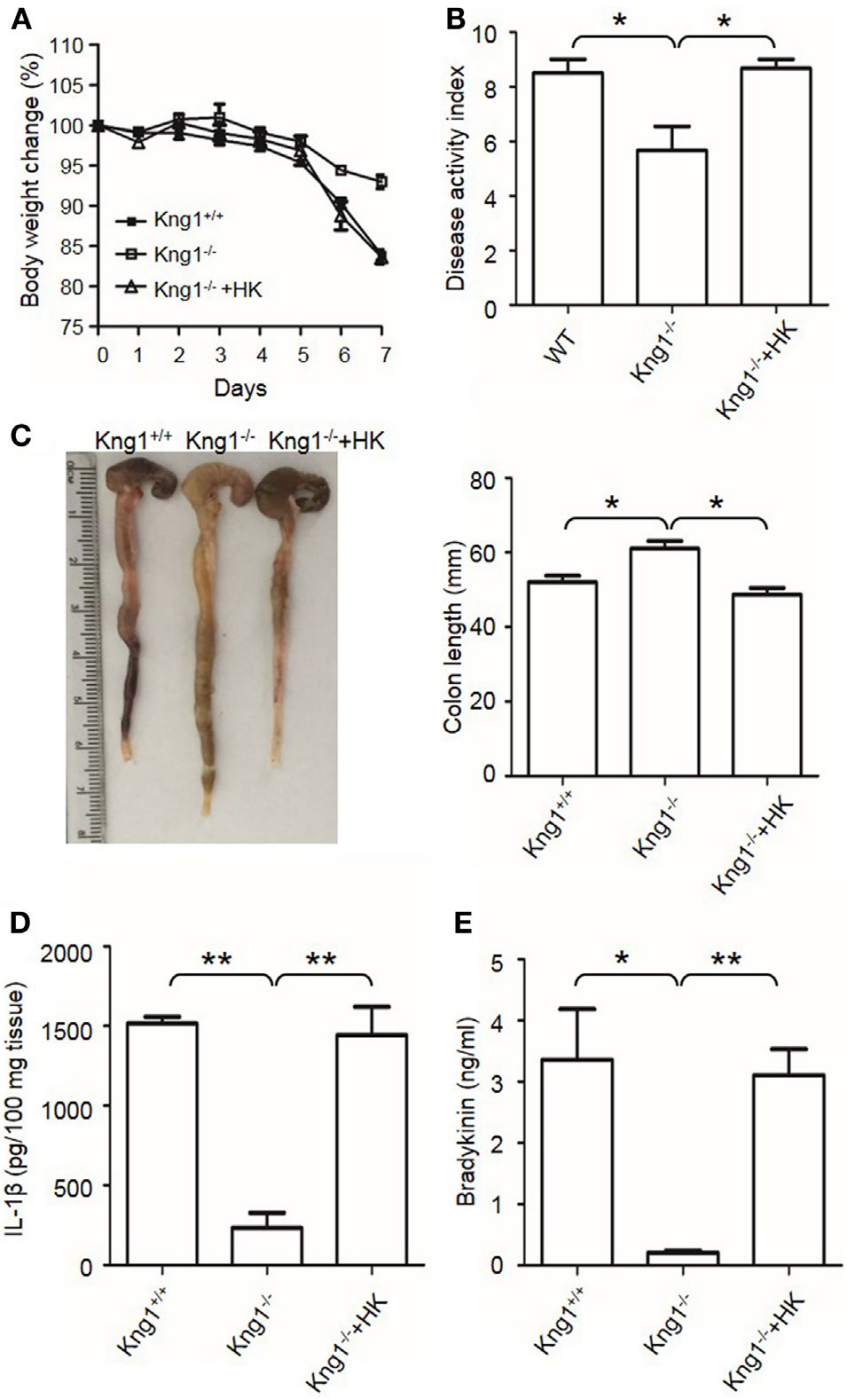
Reconstitution of *Kng1*^−/−^ mice with high-molecular-weight kininogen (HK) recovers their susceptibility to dextran sulfate sodium (DSS)-induced colitis. Three groups of *Kng1*^+/+^, *Kng1*^−/−^, and *Kng1*^−/−^ mice replenished with HK were treated with 2.5% dextran sulfate sodium (*n* = 4). On day 0, *Kng1*^−/−^ mice were injected with HK (330 μg/mouse) through the tail vein 30 min before treatment with 2.5% DSS, followed by their daily injection of HK at the same dose. Body weight change **(A)** and disease activity index **(B)** were monitored every day. The data of DAI on day 7 are shown. In **(A)**, day 6, *Kng1*^+/+^ vs. *Kng1*^−/−^: *P* < 0.01, *Kng1*^−/−^ + HK vs. *Kng1*^−/−^: *P* < 0.05; day 7, *Kng1*^+/+^ vs. *Kng1*^−/−^: *P* < 0.001, *Kng1*^−/−^ + HK vs. Kng1^−/−^: *P* < 0.01. On day 7, the mice were sacrificed and the cecum and colon were collected. The representative gross colon appearance is shown [left panel, **(C)**], and colon length was measured [right panel, **(C)**]. IL-1β levels in the colon homogenates **(D)** and bradykinin concentrations in plasma **(E)** were measured by enzyme-linked immunosorbent assay. Statistical significance was determined by one-way analysis of variance. *P < 0.05; **P < 0.01.

Finally, we tested whether the reconstitution of *Kng1*^−/−^ mice with HK recovers their susceptibility to DSS-induced colitis. Unlike *Kng1*^−/−^ mice, *Kng1*^−/−^ mice replenished with HK had severe body weight loss and DAI comparable to wild-type mice (Figures 3A,B). Reconstitution of *Kng1*^−/−^ mice with HK also resulted in shortened colon length (Figure 3C), increased IL-1β levels in the colon tissue, and increased bradykinin concentration in the plasma (Figures 3D,E). These results indicate that systemic administration of exogenous HK in *Kng1*^−/−^ mice rescues the susceptible phenotype in the DSS model, suggesting that the phenotype of *Kng1*^−/−^ mice results from the loss of HK.

### Combined Deficiency of Two Bradykinin Receptors (B1R and B2R) Attenuates DSS-Induced Colitis and Suppresses Colonic Cytokine Production and MPO Levels

**Figure 4:**
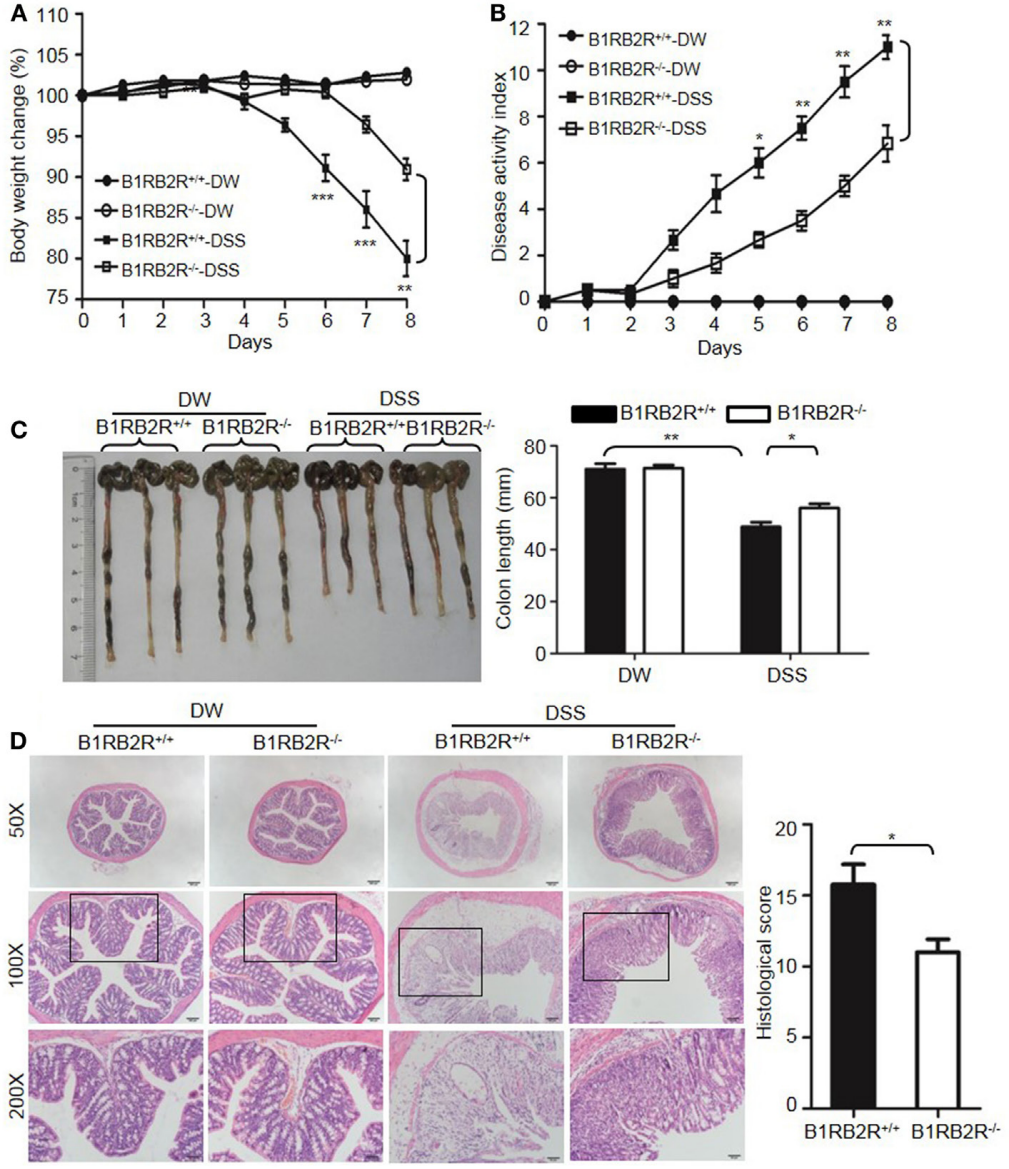
B1RB2R deficiency inhibits dextran sulfate sodium (DSS)-induced colitis. Four groups of *B1RB2R*^+/+^ mice and *B1RB2R*^−/−^ mice were treated with distilled water (DW) or 2.5% DSS for 8 days (*n* = 12). Body weight change **(A)** and disease activity index **(B)** were monitored every day. **(C)** On day 8, the cecum and colon were isolated. The representative gross appearance of the colons (left) is shown. Colon length (right) was measured. **(D)** Representative H&E staining (left panel) is shown. Histological scoring was calculated (right panel). Statistical significance was determined by one-way analysis of variance **(A,C)** and Mann–Whitney test **(B,D)**: *P < 0.05; **P < 0.01; ***P < 0.001.

**Figure 5:**
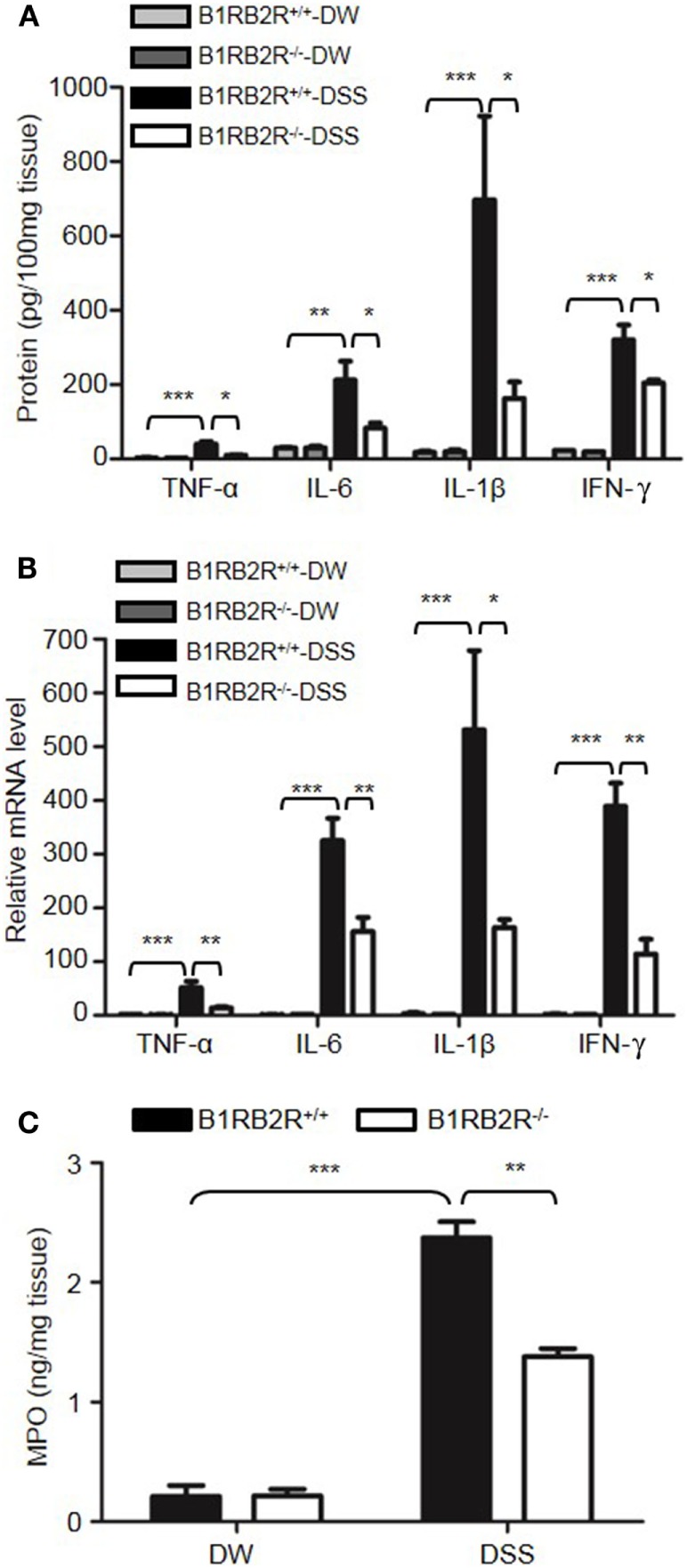
B1RB2R deficiency inhibits cytokine production. Four groups of *B1RB2R*^+/+^ mice and *B1RB2R*^−/−^ mice were treated with distilled water (DW) or 2.5% dextran sulfate sodium (DSS) for 8 days (*n* = 12). **(A)** Cytokine concentrations in the colon homogenates were measured by enzyme-linked immunosorbent assay (ELISA). **(B)** RNA was isolated from the colons and cytokine mRNA expression was measured by real-time RT-PCR. **(C)** Myeloperoxidase (MPO) concentrations in colon homogenates were measured by ELISA. Statistical significance was determined by one-way analysis of variance: *P < 0.05; **P < 0.01; ***P < 0.001.

Upon activation of the KKS, HK is cleaved to release bradykinin. Because HK deficiency inhibited DSS-induced colonic inflammation (Figures 1 and 2), we determined the role of the bradykinin receptors in mice lacking both B1 and B2 bradykinin receptors (*B1RB2R*^−/−^). Although *B1RB2R*^+/+^ mice displayed severe colitis over the 8 days of DSS treatment, *B1RB2R*^−/−^ mice had a delayed onset and a noticeably milder course of disease, as evidenced by their significantly lower body weight loss and DAI (Figures 4A,B). While *B1RB2R*^+/+^ mice experienced significant colonic shortening and severe destructive colitis, these changes were significantly attenuated in *B1RB2R*^−/−^ mice (Figures 4C,D). Consistently, IFN-γ, TNFα, IL-1β, and IL-6 protein and mRNA levels in the colon homogenate were markedly increased in DSS-treated *B1RB2R*^+/+^ mice, whereas they were significantly downregulated in *B1RB2R*^−/−^ mice (Figures 5A,B). In addition, DSS treatment increased MPO levels in colon homogenates from *B1RB2R*^+/+^ mice, the levels were markedly reduced in *B1RB2R*^−/−^ mice (Figure 5C). These results suggest that bradykinin generated from HK is important for the pathogenesis of DSS-induced colitis.

### pKal but Not FXII Is Required for DSS-Induced Colitis

**Figure 6:**
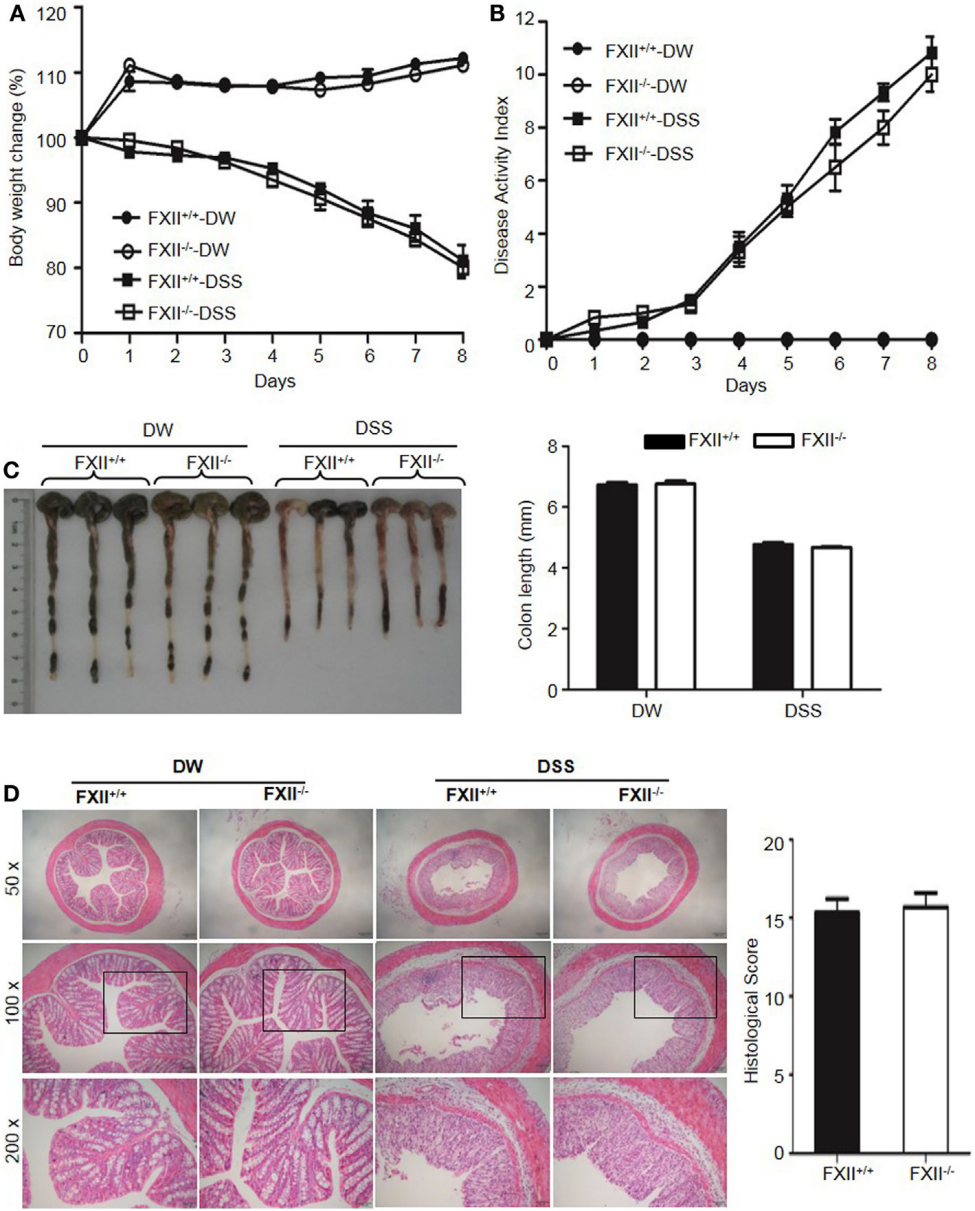
Factor XII (FXII) deficiency does not affect dextran sulfate sodium (DSS)-induced colitis. Four groups of *FXII*^+/+^ mice and *FXII*^−/−^ mice were treated with distilled water (DW) or 2.5% DSS for 8 days (*n* = 12). Body weight change **(A)** and disease activity index **(B)** were monitored every day. **(C)** On day 8, the cecum and colon were isolated. Representative gross appearance of colons (left) is shown. Colon length (right) was measured. **(D)** Representative H&E staining (left panel) is shown. Histological scoring was calculated (right panel). Statistical significance was determined by one-way analysis of variance **(A,C)** and Mann-Whitney test **(B,D)**.

The above observations suggested that KKS activation is involved in DSS-induced colitis. We, therefore, tested whether FXII and/or pKal are upstream of HK. First, we examined the phenotype of *FXII*^−/−^ mice. In contrast to the protective phenotypes of *Kng1*^−/−^ and *B1RB2R*^−/−^ mice, the weight loss (Figure 6A), DAI scores (Figure 6B), colon shortening (Figure 6C), and histological changes (Figure 6D) in *FXII*^−/−^ mice were comparable to those of WT mice, indicating that FXII is not involved in the pathogenesis of DSS-induced colitis.

**Figure 7:**
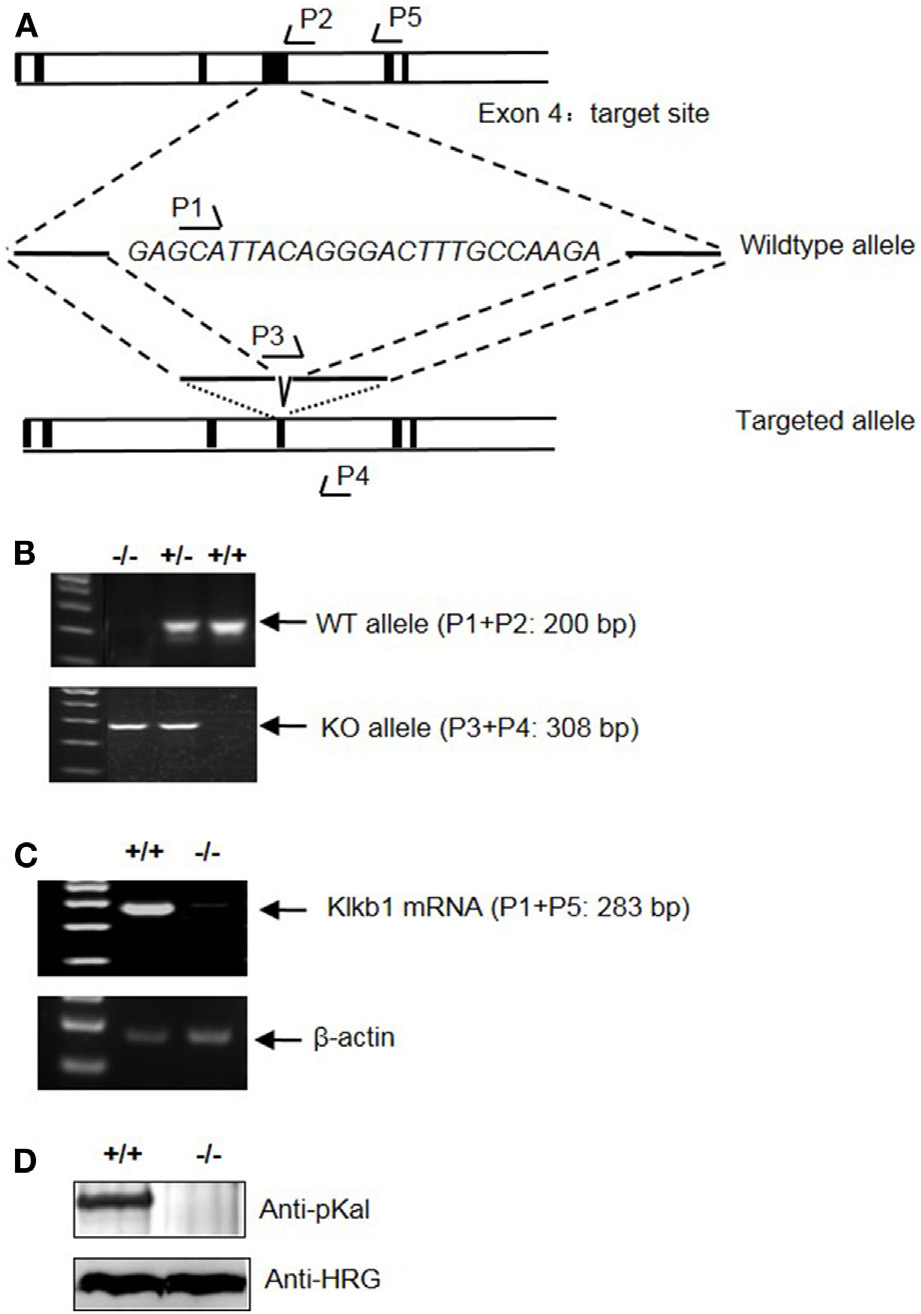
Generation and characterization of *Klkb1*^−/−^ mice. **(A)** Schematic design of the mouse Klkb1 gene deletion. Exon 4 of Klkb1 gene was selected as the transcription activator-like effector nucleases target site. PCR primer (P1, P2, P3, P4, and P5) positions are shown. Sequencing confirmed the deletion of 25 bp (GAGCATTACAGGGACTTTGCCAAGA; 243–267 of the ORF) in the knockout allele. **(B)** PCR of the genomic DNA yielded a 200-bp product for the WT allele using primer pairs P1 and P2, and a 308-bp product for the knockout allele using primer pairs P3 and P4. **(C)** Expression of *Klkb1* mRNA in the liver. Total RNA was isolated from the liver of WT mice and *Klkb1*^−/−^ mice. The expression of *Klkb1* mRNA was measured by RT-PCR using primer pairs P1 and P5. β-Actin mRNA served as a loading control. **(D)** Expression of prekallikrein (pKal) antigen in the plasma was detected by immunoblotting with an anti-pKal antibody with histidine-rich glycoprotein as a loading control.

To determine the role of pKal in DSS-induced colitis, we generated a mouse strain with a disrupted *Klkb1* gene, which encodes pKal (*Klkb1*^−/−^ mice). Exon 4 of the mouse *Klkb1* gene was selected as the TALEN targeting site (Figure 7A). Genotyping (Figure 7B) and sequencing results (data not shown) confirmed a 25-bp deletion (GAGCATTACAGGGACTTTGCCAAGA; 243–267 of the open reading frame) in the targeted allele. *Klkb1* mRNA expression was absent in the *Klkb1*^−/−^ mouse livers (Figure 7C). Moreover, the pKal antigen was not detected in the *Klkb1*^−/−^ mouse plasma (Figure 7D).

**Figure 8:**
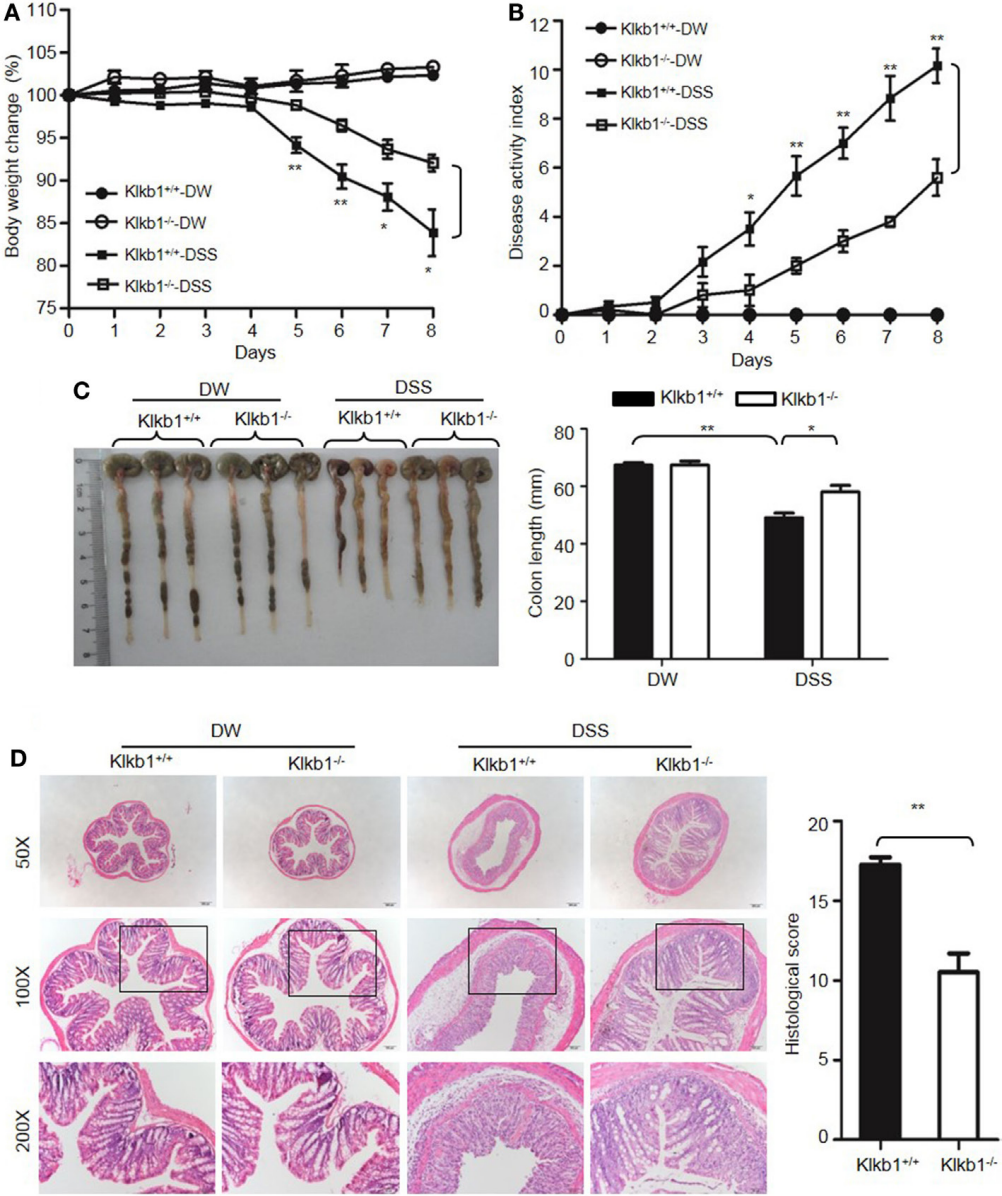
Prekallikrein deficiency attenuates dextran sulfate sodium (DSS)-induced colitis. Four groups of *Klkb1*^+/+^ mice and *Klkb1*^−/−^ mice were treated with distilled water (DW) or 2.5% DSS for 8 days (*n* = 12). Body weight change **(A)** and disease activity index **(B)** were monitored every day. **(C)** On day 8, the cecum and colon were isolated. Representative gross appearance of the colons (left) is shown. Colon length (right) was measured. **(D)** Representative H&E staining (left panel) is shown. Histological scoring was calculated (right panel). Statistical significance was determined by one-way analysis of variance **(A,C,D)** and Mann–Whitney test **(B)**: *P < 0.05; **P < 0.01; ***P < 0.001.

**Figure 9:**
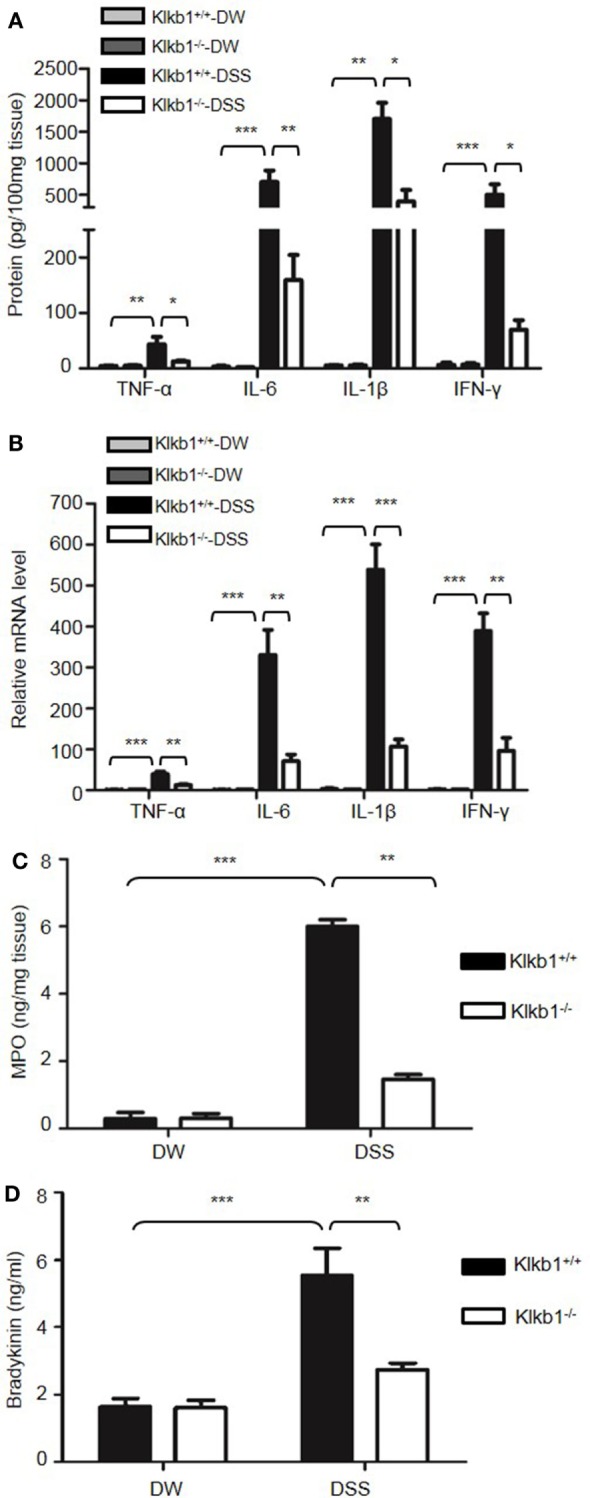
Prekallikrein deficiency decreases the production of cytokines and bradykinin. Four groups of *Klkb1*^+/+^ mice and *Klkb1*^−/−^ mice were treated with distilled water (DW) or 2.5% dextran sulfate sodium (DSS) for 8 days (*n* = 12). **(A)** Cytokine concentrations in colon homogenates were measured by enzyme-linked immunosorbent assay (ELISA). **(B)** RNA was isolated from the colons, and the cytokine mRNA expression was measured by real-time RT-PCR. **(C)** Myeloperoxidase (MPO) concentrations in colon homogenates were measured by ELISA. **(D)** Bradykinin concentration in the plasma was measured by ELISA. Statistical significance was determined using one-way analysis of variance: *P < 0.05; **P < 0.01; ***P < 0.001.

After treatment with DSS, *Klkb1*^−/−^ mice showed a significant decrease in body weight loss and DAI (Figures 8A,B), and exhibited significant attenuation in colon shortening and destructive colitis (Figures 8C,D) compared to *Klkb1*^+/+^ mice. Cytokine concentrations were evaluated in DSS-treated *Klkb1*^+/+^ mice and *Klkb1*^−/−^ mice. The protein levels of major cytokines, including TNFα, IL-6, IL-1β, and IFN-γ, were significantly increased in the colon homogenates of DSS-treated *Klkb1*^+/+^ mice compared to those in DW-treated mice; however, these cytokine levels significantly decreased in DSS-treated *Klkb1*^−/−^ mice (Figure 9A). The mRNA levels of these cytokines in colon homogenates also significantly decreased in DSS-treated *Klkb1*^−/−^ mice compared to those in *Klkb1*^+/+^ mice (Figure 9B). Additionally, MPO levels were lower in the colon tissue of DSS-treated *Klkb1*^−/−^ mice than in *Klkb1*^+/+^ mice (Figure 9C). Bradykinin concentration also significantly decreased in DSS-treated *Klkb1*^−/−^ mice compared to those in DSS-treated *Klkb1*^+/+^ mice (Figure 9D). These results suggest that pKal is important in the pathogenesis of DSS-induced colitis and is responsible for the production of bradykinin.

### Inhibition of Kal Activity Ameliorates DSS-Induced Colitis

**Figure 10:**
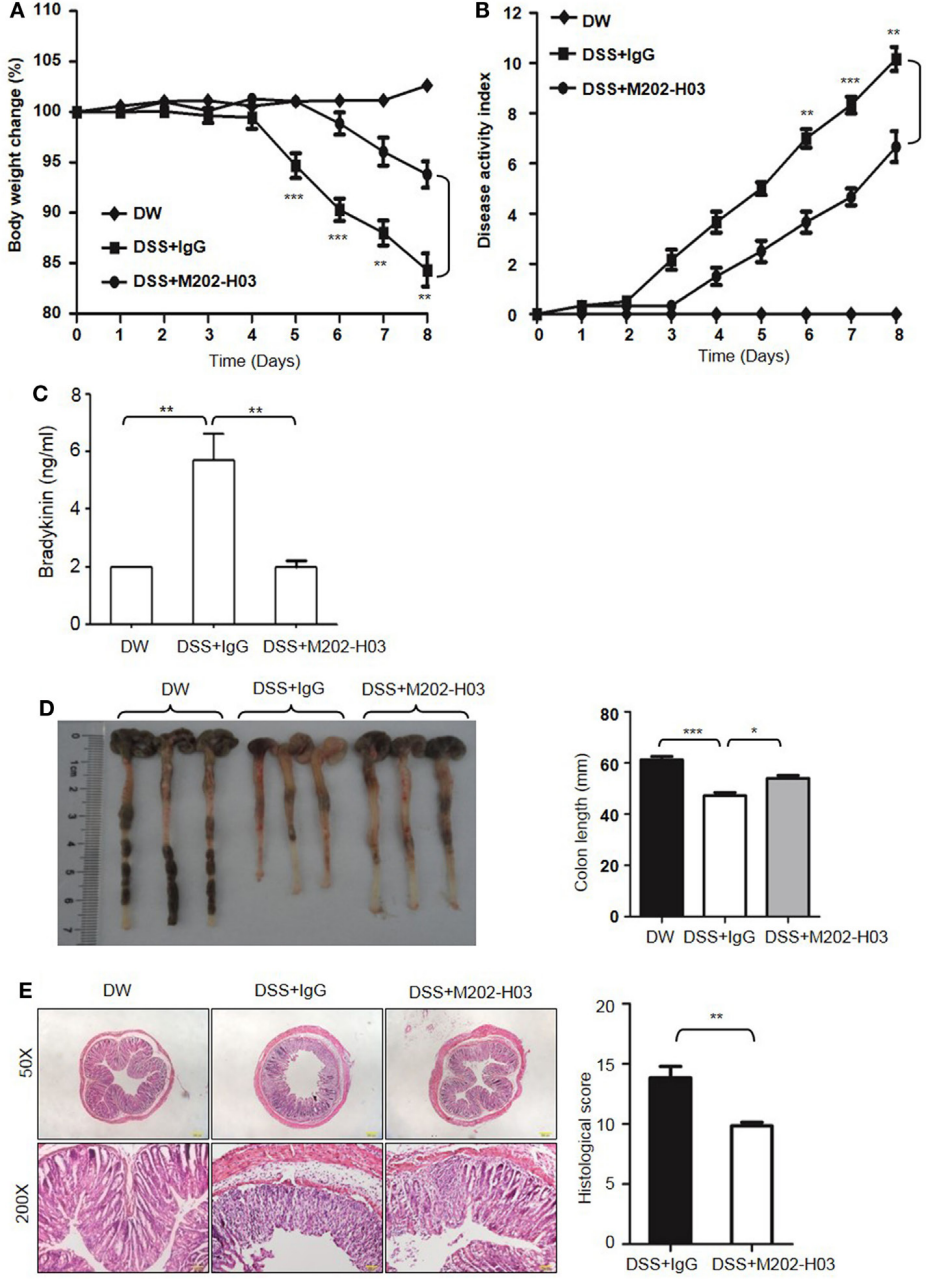
Inhibition of Kal ameliorates dextran sulfate sodium (DSS)-induced colitis. WT C57BL/6 mice were randomly divided into three groups (*n* = 6), receiving treatment with distilled water (DW), 2.5% DSS in drinking water plus intraperitoneal injection of 20 mg/kg of control IgG1, or 2.5% DSS in drinking water plus intraperitoneal injection of 20 mg/kg of M202-H03, for 8 days. Body weight change **(A)** and disease activity index **(B)** were monitored every day. On day 8, the mice were sacrificed and the blood, cecum, and colon were collected. Bradykinin concentration in plasma was measured by enzyme-linked immunosorbent assay **(C)**. Representative gross appearance of the colon is shown [left panel, **(D)**] and colon length was measured [right panel, **(D)**]. **(E)** The distal colon tissue were fixed and the paraffin sections were processed for hematoxylin and eosin staining, with a representative image (left panel) and histological scores (right panel) shown here. Statistical differences were determined by one-way analysis of variance **(A–D)** and *t*-test **(E)**: *P < 0.05, **P < 0.01, ***P < 0.001.

To examine whether Kal activity is involved in DSS-induced colitis, WT C57BL/6 mice were treated with a highly specific active site-blocking antibody (M202-H03) to inhibit Kal *in vivo* (40). M202-H03 treatment significantly inhibited DSS-induced colitis, while treatment with an isotype-matched control IgG had little effect (Figures 10A,B). Moreover, M202-H03 treatment significantly decreased plasma bradykinin levels (Figure 10C). Compared with isotype-matched control IgG, M202-H03 significantly increased the colon length (Figure 10D), ameliorated the loss of epithelial integrity and infiltration of inflammatory cells, and decreased the histological score (Figure 10E). These data suggest that Kal activity is important in the pathogenesis of DSS-induced colitis and support a functional connection between pKal, HK and bradykinin.

### Kal Is Required for Increased Generation of Bradykinin in the Plasma of DSS-Treated Mice

**Figure 11:**
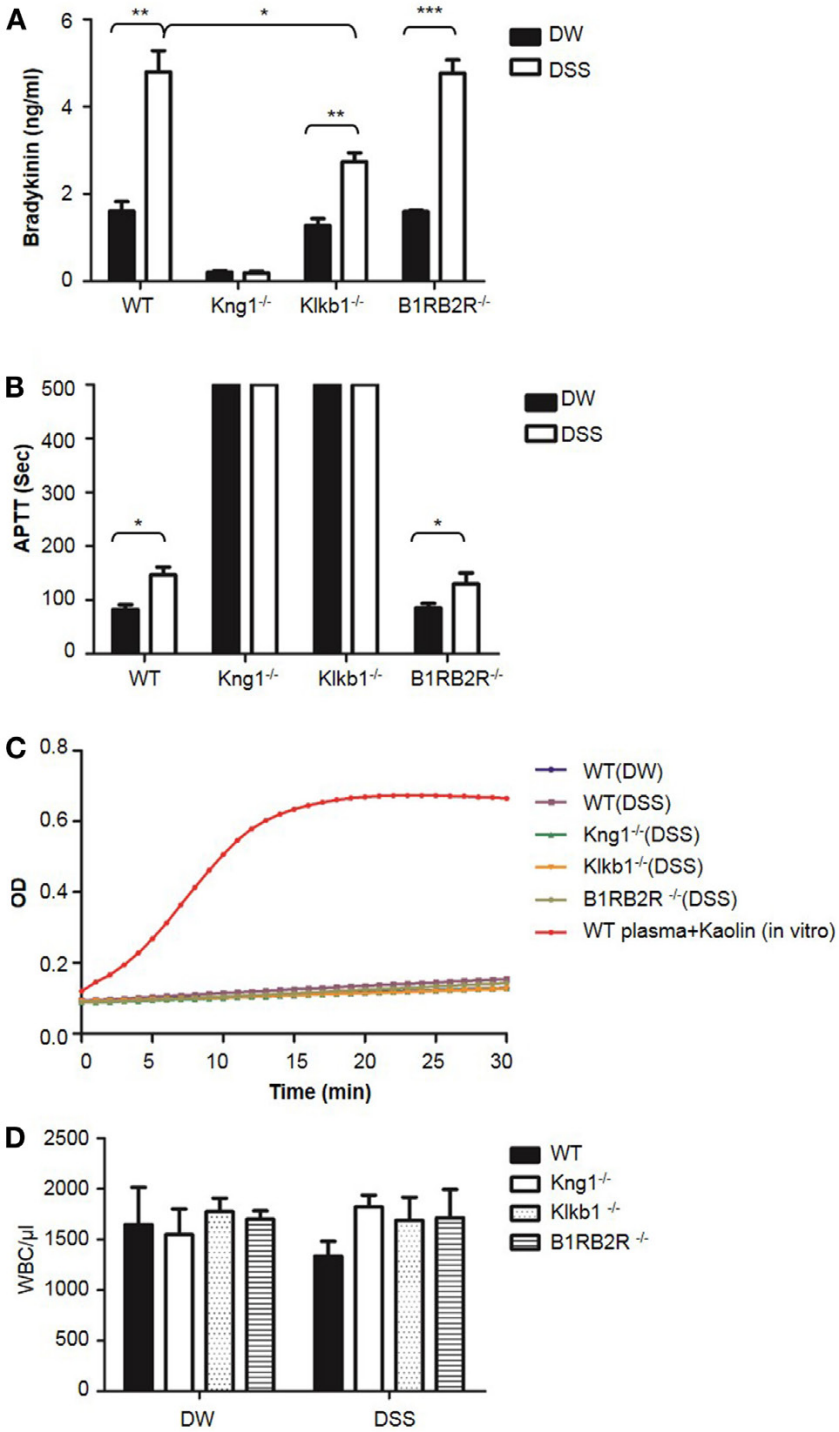
Comparison of white blood cell count, bradykinin levels, APTT, and FXIIa activity in peripheral blood among WT, *Kng1*^−/−^, *Klkb1*^−/−^, and *B1RB2R*^−/−^ mice. WT C57BL/6 mice, *Kng1*^−/−^, *Klkb1*^−/−^, and *B1RB2R*^−/−^ mice were treated with distilled water (DW) or 2.5% dextran sulfate sodium (DSS) (*n* = 6). On day 8, the mice were sacrificed and blood was collected. Bradykinin levels **(A)**, APTT **(B)**, FXIIa activity **(C)**, and white blood cell counts **(D)** were measured. In **(C)**, FXIIa activity in the plasma from WT C57BL/6 mice treated with 100 µg/mL of Kaolin *in vitro* served as a positive control. Statistical significance was determined by one-way analysis of variance. *P < 0.05; **P < 0.01; ***P < 0.001.

To understand the protective phenotypes observed in *Kng1*^−/−^, *Klkb1*^−/−^, and *B1RB2R*^−/−^ mice in DSS-induced colitis, we measured bradykinin levels in the plasma which was prepared using EDTA as an anticoagulant. WT and *B1RB2R*^−/−^ mice had a significant increase in bradykinin levels on day 8 after DSS treatment indicating activation of the KKS (Figure 11A). In contrast, DSS treatment did not yield bradykinin production in plasma of *Kng1*^−/−^ mice (Figure 11A). *Klkb1*^−/−^ mice had significantly lower levels of bradykinin than WT mice (Figure 11A), suggesting that pKal is required for DSS-induced bradykinin production. Consistent with the observed increase in bradykinin levels, WT and *B1RB2R*^−/−^ mice also showed significantly prolonged APTT upon DSS treatment (Figure 11B). As expected, prolonged APTT was detected in both *Kng1*^−/−^ and *Klkb1*^−/−^ mice, with or without DSS treatment (Figure 11B). We measured FXIIa activity in these mice and found no increase in FXIIa activity in the plasma (Figure 11C), suggesting that FXII activation was not detectable in this disease model. Furthermore, we found that peripheral white blood cell numbers were comparable among these mice (Figure 11D), suggesting that at late stage colitis, the KKS does not modulate circulating inflammatory cell numbers (Figure 2D).

### DSS Activates pKal to Induce Cleavage of HK and Release Bradykinin

**Figure 12:**
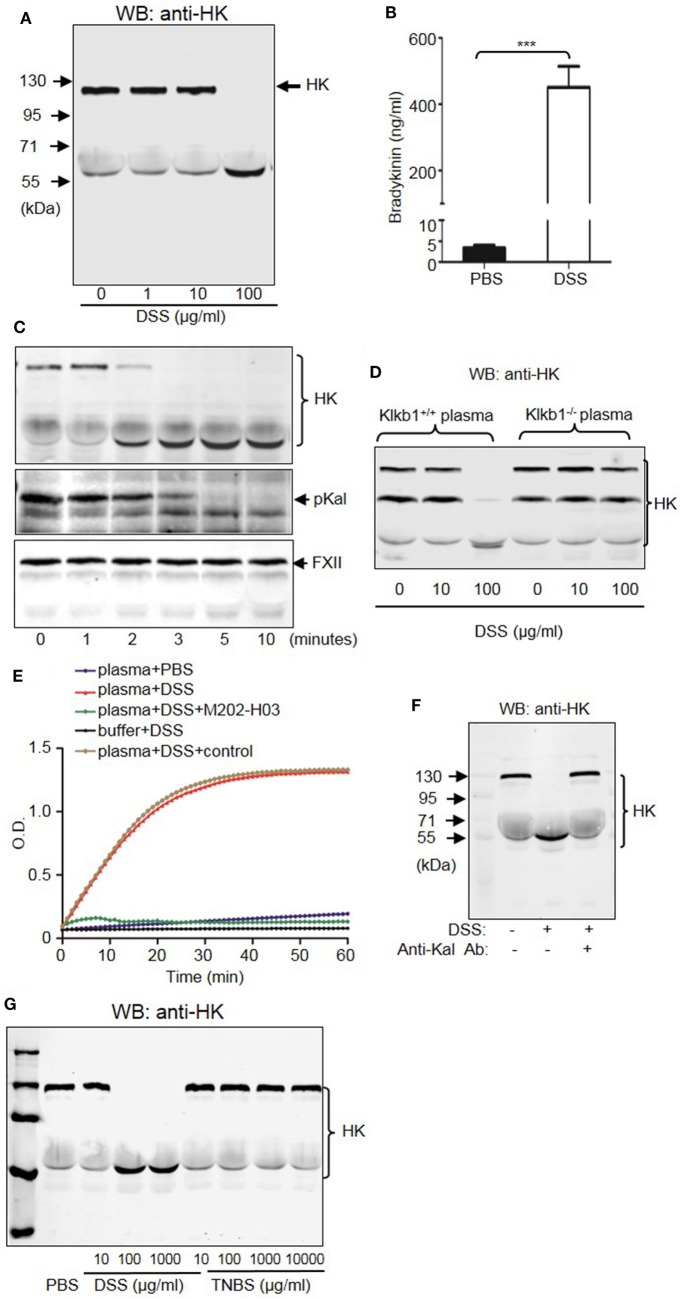
Dextran sulfate sodium (DSS) induces activation of the kallikrein–kinin system (KKS). **(A)** DSS-initiated high-molecular-weight kininogen (HK) cleavage in the plasma. Human platelet-free plasma was incubated with DSS at the indicated concentrations for 30 min at 37°C. Cleavage of HK was detected *via* immunoblotting. **(B)** Human plasma was incubated with PBS (control) and 100 µg/mL of DSS at 37°C for 30 min. The production of bradykinin was measured *via* enzyme-linked immunosorbent assay. **(C)** Time course of DSS-triggered KKS activation in the plasma. Human plasma was incubated with 100 µg/mL of DSS for the indicated times. Proteins were separated by reducing SDS-PAGE and detected *via* immunoblotting using antibodies for HK (upper panel), prekallikrein (pKal) (middle panel), and factor XII (FXII) (lower panel). **(D)** Plasma from *Klkb1*^+/+^ mice and *Klkb1*^−/−^ mice was incubated with DSS at the indicated concentrations for 30 min. Cleavage of HK was detected by immunoblotting using a polyclonal anti-HK antibody. **(E)** Human plasma was incubated in a total volume of 100 µL with PBS, 100 µg/mL of DSS, 100 µg/mL of DSS plus 30 µg/mL of anti-Kal antibody M202-H03, or 100 µg/mL of DSS plus 30 µg/mL of isotype-matched IgG1. Buffer plus 100 µg/mL of DSS was used as a control. Cleavage of the chromogenic substrate S-2302 indicated Kal activity, and was measured by reading the absorbance at 405 nm for 60 min. **(F)** Human plasma was incubated with or without 100 µg/mL of DSS in the presence of 30 µg/mL of anti-Kal antibody, or its isotype-matched control IgG1. Cleavage of HK was detected by immunoblotting by using a polyclonal anti-HK antibody. **(G)** Human plasma was incubated with DSS or TNBS at the indicated concentrations at 37°C for 30 min. Cleavage of HK was detected by immunoblotting using a polyclonal anti-HK antibody. The above experiments were repeated three times and representative images of the blots are shown here.

To understand the mechanism by which the KKS contributes to DSS-induced colitis, we examined whether DSS induced HK cleavage as a function of concentration and time. According to immunoblotting, 100 µg/mL DSS induced complete HK cleavage in human plasma (Figure 12A). Consistently, DSS increased bradykinin production by more than 100-fold compared to PBS (Figure 12B). Activation of serine proteases like pKal depends on cleavage. We found that DSS induced cleavage of both pKal and HK in plasma as early as 2 min after incubation, without inducing FXII cleavage (Figure 12C). To determine whether DSS induced HK cleavage *via* pKal, we compared DSS-induced HK cleavage in the presence and absence of pKal. DSS induced the cleavage of HK in *Klkb1*^+/+^ mouse plasma, but not in *Klkb1*^−/−^ mouse plasma (Figure 12D), demonstrating that pKal is required for DSS-induced HK cleavage. Furthermore, we determined that HK cleavage by DSS is dependent on the activation of Kal using a specific anti-Kal antibody, M202-H03 (Ki < 1 nM) (40). In our previous study, we found that the anti-Kal antibody significantly inhibited Kal activation in rodents (29). In the present study, we used a chromogenic assay to demonstrate that DSS induced the activation of pKal, and that this induction was prevented by the anti-Kal antibody, but not its isotype-matched control IgG1 (Figure 12E). Moreover, DSS-induced HK cleavage in the plasma was completely blocked by the anti-Kal antibody (Figure 12F). These data suggest that activated pKal cleaves HK to release bradykinin, implicating this pathway in the pathogenesis of DSS-induced colitis. HK preferentially binds to negatively charged molecules in a complex with pKal. Since DSS is a negatively charged polymer of glucose with engrafted sulfate groups, it could be associated with HK, which may account for the assembly of the HK–pKal complex with DSS polymers, and subsequent activation of pKal.

### The pKal-HK Pathway Is also Important in the Pathogenesis of TNBS-Induced Colitis

**Figure 13:**
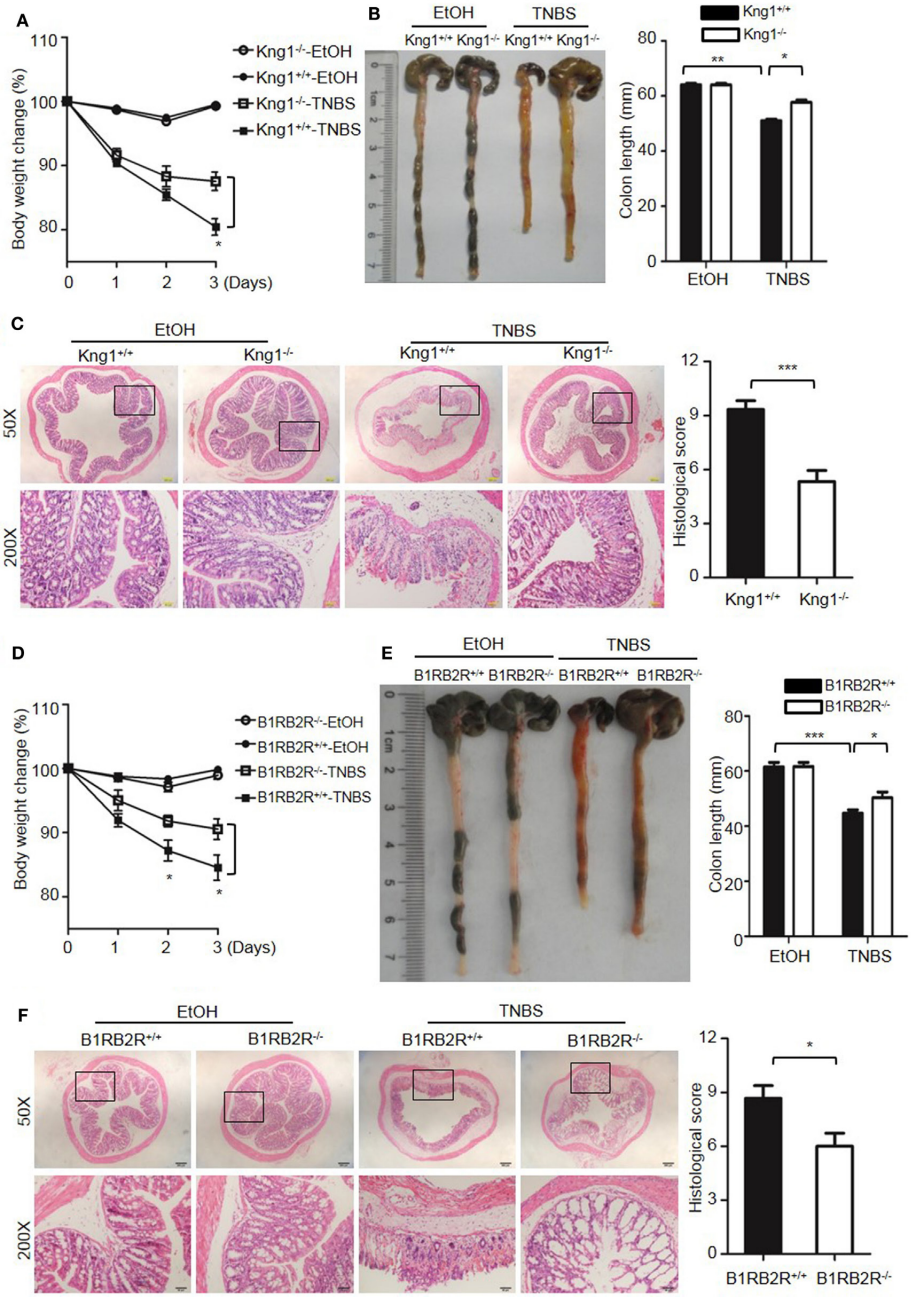
Deficiency of high-molecular-weight kininogen or B1RB2R ameliorates TNBS-induced colitis. Four groups of *Kng1*^+/+^ mice and *Kng1*^−/−^ mice **(A–C)**, and four groups of B1RB2R^+/+^ mice and B1RB2R^−/−^ mice **(D–F)** were treated with EtOH or TNBS (*n* = 12). **(A,D)** Percentage body weight changes were monitored daily. **(B,E)** Gross appearance of colons (left) and reduction in colon length (right) were examined. **(C,F)** Representative H&E staining and histopathological scoring of the colons are shown. Statistical significance was determined by one-way analysis of variance **(A–E)** or Mann–Whitney test **(F)**: *P < 0.05; **P < 0.01; ***P < 0.001.

**Figure 14:**
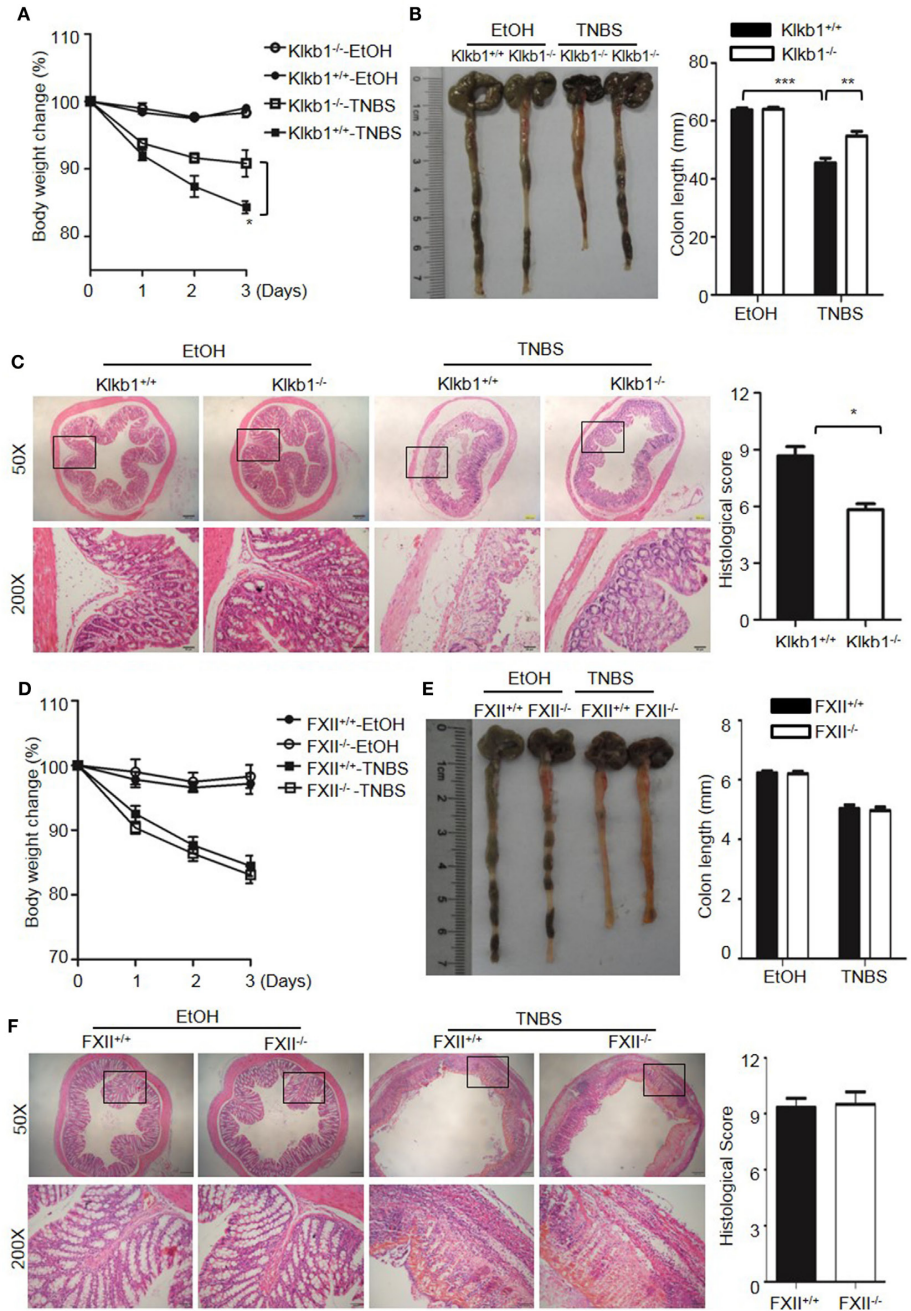
Deficiency of prekallikrein, but not factor XII (FXII), ameliorates TNBS-induced colitis. Four groups of Klkb1^+/+^ mice and Klkb1^−/−^ mice **(A–C)**, and four groups of FXII^+/+^ mice and FXII^−/−^ mice **(D–F)** were treated with EtOH or TNBS (*n* = 12). **(A,D)** Percentage body weight changes were monitored daily. **(B,E)** Gross appearance of colons (left) and reduction in colon length (right) were examined. **(C,F)** Representative H&E staining and histopathological scoring of the colons are shown. Statistical significance was determined by one-way analysis of variance: *P < 0.05; **P < 0.01; ***P < 0.001.

Because DSS activates the KKS, we evaluated whether the role of KKS in colitis is a model-dependent event. Similarly to DSS, TNBS can induce colitis in mice. However, unlike DSS, TNBS did not induce HK cleavage, even at a concentration of 10,000 µg/mL (Figure 12G), indicating that TNBS does not activate the KKS. Similar to our DSS model, we investigated the phenotypes of mice lacking KKS components in TNBS-induced colitis. *Kng1*^−/−^ mice (Figures 13A–C), *B1RB2R*^−/−^ mice (Figures 13D–F), or *Klkb1*^−/−^ mice (Figures 14A–C) had significant protection against TNBS-induced colitis, as evidenced by the amelioration of body weight loss, colon length shortening, and histological changes. However, similar to the phenotype observed in DSS-induced colitis, *FXII*^−/−^ mice developed disease like *FXII*^+/+^ mice in the TNBS model (Figures 14D–F). These data support the important role of the pKal-HK pathway in the general mechanism of colitis.

## Discussion

This is the first comprehensive genetic analysis of the role of KKS in IBD. Using KKS-knockout mice in two experimental colitis models, the present study demonstrates that pKal, HK, or combined B1R/B2R, but not FXII, deficiency protects against colitis. The study provides the first genetic confirmation of the critical importance of pKal-HK pathway in the pathogenesis of colitis in these models. The role for HK in colitis development is likely dependent on bradykinin production, as a similar phenotype was observed in B1RB2R-deficient mice. Whether HK also contributes to colitis pathogenesis through other mechanisms (such as enhancement of endotoxin activity) awaits further investigation.

The mechanism that triggers the pKal-HK pathway and may contribute to colonic inflammation can only be speculated upon. Previous studies have shown that the initial step of KKS activation is mediated by the binding of HK to an activation surface (3, 4), made up of negatively charged molecules, such as nucleic acids, oversulfated chondroitin sulfate, polyphosphate, collagen, misfolded protein aggregates, lipopolysaccharides, and glycosaminoglycans (3). Thus, HK not only binds to molecules released from necrotic cells such as nucleic acids and polyphosphate but also to phosphatidylserine from apoptotic cell membrane. In addition, in the lumen of the inflamed colon, HK may bind to bacteria and exuded subendothelial extracellular matrix proteins, leading to activation of the KKS (41). We recently showed that pKal was activated during reactive arthritis induced by peptidoglycan–polysaccharide, and that the pKal-HK pathway is involved in this process (29).

Normally, approximately 75% of pKal circulates in the plasma and is bound noncovalently to HK at a 1:1 ratio (3). Presumably, binding of HK to negatively charged molecules and/or apoptotic/necrotic cells in the local colonic tissue may enrich the pKal–HK complex at this site. Consequently, pKal can be activated to Kal, which cleaves HK to release bradykinin. Bradykinin is a potent mediator of vascular permeability and vasodilatation, and also a major spasmogen of the smooth muscle of the gut (19). This could explain why HK is needed for the recruitment of pro-inflammatory neutrophils and monocytes into the inflamed colon (Figure 3). Through its two receptors, B1R and B2R, bradykinin induces inflammatory cytokines including IL-1β (11, 29, 42), and increases the levels of a variety of other pro-inflammatory factors such as nitric oxide, prostaglandins, leukotrienes, platelet-activating factor, and neuropeptides (3, 43). A role for IL-1 β has previously been suggested in the development of enterocolitis (44–46). The bradykinin receptors, B1R and B2R, are expressed in intestines of both healthy individuals and patients with IBD, but their expression is significantly increased in intestines from patients with active IBD (16). Presumably, once bradykinin is generated following activation of the KKS, it mediates plasma exudation, resulting in a supply of KKS proteins at the inflammatory sites. This positive feedback loop may occur at inflammatory sites, possibly explaining the role of bradykinin in the pathogenesis of IBD. We found that whereas simultaneous deficiency in both bradykinin receptors inhibit colitis, previous studies found that B2R deficiency alone is not sufficient to protect from colitis in the DSS model (47). This suggests an involvement of B1R. However, because of the dynamic expression patterns during disease development, the specific role of the two bradykinin receptors in the pathogenesis of IBD is complex, and additional studies are warranted to evaluate their dynamic cooperation and distinct involvement.

The phenotype of pKal-deficient mice in the two colitis models studied here suggests that Kal contributes to the general mechanism of colitis, which is consistent with the observed ameliorative effect of a Kal inhibitor in an IBD model (13). The release of pKal in the intestinal extracellular space resulting from extravasation of blood is correlated with the degree of tissue inflammation (24). Although the major role of Kal is to generate bradykinin, it may also trigger other pathways. For example, Kal can regulate cell signaling by cleaving and activating G protein-coupled proteinase-activated receptors, thereby triggering responses such as vasodilatation, intestinal inflammation, increased cytokine production, and increased nociception (48–50). In addition, Kal itself exhibits chemotactic activity and stimulates the release of neutrophil elastase (51). Although pKal and HK are necessary for colitis pathogenesis, as evidenced by the phenotypes of mice lacking these two genes, we find that FXII is not. There are several mechanisms that may trigger FXII-independent pKal activation leading to bradykinin release from HK. On the cell surface, the lysosomal enzyme prolylcarboxypeptidase can activate pKal in an FXII-independent manner (6). Alternatively, in FXII-deficient plasma, depletion of C1 inhibitor auto-activates pKal-HK complex to release bradykinin (8). In addition, Kal can be activated by heat shock protein 90, which is released from endothelial cells (8, 9). However, the mechanism of FXII-independent activation of pKal in the pathogenesis of colitis requires further investigation. Although FXII presumably does not play a role in the models described here, it may still be involved in other cases. Crohn’s disease, UC, celiac disease, irritable bowel syndrome, and food allergies have been associated with mast cell hyperplasia in the mucosa and humoral activity (52, 53). In mouse models of allergic lung inflammation and *Trypanosoma cruzi* infection, mast cells release cellular contents such as histamine and polyphosphate that activate FXII, linking mast cell degranulation to FXII-dependent bradykinin release (54, 55).

The phenotypes of B1RB2R-deficient mice suggest that bradykinin and/or des(Arg9)-kinin are the downstream effector molecules involved in the immunopathology in the classical models of DSS or TNBS-induced colitis. These potent peptides, once released in the injured/inflamed mucosa, might modulate the function of a wide range of immune cells, such as neutrophils, macrophages, innate lymphoid cells, dendritic cells (DCs), and T cells (16, 19). Macrophages, DCs, and T cells express GPCRs, including B1R, B2R and PARs (56), but little is known about their role in the intestinal mucosa. In addition, these GPCRs are also expressed by epithelial cells, neurons, and endothelium (16). Because innate immunity is intertwined with neuronal networks (57), the released kinins could play a role in neuro-immune interactions driving colitis development. To further unravel the full complexity of the KKS network in mucosal immunity will be a challenge, and conditional knockout mouse models will be necessary for delineation of the specific role of B1R and B2R in different cell types during the development of colitis.

We recently reported that deficiency of HK, but not pKal or FXII, resulted in protection against endotoxin-induced lethality in mice, suggesting that different KKS proteins play distinct roles in host response to endotoxin (22). The different phenotypes of KKS-deficient mice between endotoxin-induced sepsis and DSS/TNBS-induced colitis probably result from distinct function of the KKS in different disease models. It has been reported that DSS and TNBS increase translocation of the colon-associated microbiota from the gut lumen into the mucosa (58–60). These reagents may hence promote microbial translocation and thereby activate innate immunity receptors to initiate inflammation which leads to plasma leakage into the mucosal tissues. There is also a possibility that the resistant phenotype of *Kng1*^−/−^ mice and the susceptibility of *Kng1*^−/−^ mice reconstituted with HK may result from the binding of HK to endotoxin displayed by subsets of bacterial pathogens translocated from the gut and subsequent activation of inflammatory cells *via* the HK/toll-like receptor (TLR4) pathway. Moreover, following such leakage, pKal, BK, des-Arg9-BK and B2/B1R might further amplify/propagate the inflammation. TLR4 and MD2 are indeed upregulated in colonic tissue during DSS-induced inflammation (61), but if HK is also associated with increased levels of endotoxin and enhance this activation awaits further investigation.

In conclusion, we demonstrated a critical role for the pKal–HK pathway in the pathogenesis of colitis in mouse IBD models. In spite of the difference in the activation of the KKS by DSS and TNBS, both reagents may damage the intestinal barrier integrity, and hence similar phenotypes of the KKS-deficient mice were observed in both models. Our finding offer novel insights into the mechanism underlying IBD pathogenesis and may also provide new prognostic and diagnostic parameters for evaluating IBD severity. Moreover, the pathways controlling KKS activation may lead to new therapeutic targets in the treatment of IBD.

## Ethics Statement

This study was carried out in accordance with protocols approved by the Institutional Animal Care and Use Committees of Soochow University and Temple University.

## Author Contributions

BW, AY, ZZ, CH, YL, JD, and YW designed and performed the experiments and collected and analyzed data. RC contributed critical reagents and helped with data interpretation. YW conceived the study, supervised the research, analyzed the data, and wrote the manuscript.

## Conflict of Interest Statement

The authors declare that the research was conducted in the absence of any commercial or financial relationships that could be construed as a potential conflict of interest.
